# Health surveillance and development of children with congenital Zika Virus syndrome: an integrative literature review

**DOI:** 10.1590/1984-0462/2022/40/2020335

**Published:** 2021-07-07

**Authors:** Fernanda de Brito Matiello, Jeniffer Stephanie Marques Hilário, Ellen Cristina Gondim, Darci Neves Santos, Débora Falleiros de Mello

**Affiliations:** a Nursing School of Ribeirão Preto, Universidade de São Paulo, Ribeirão Preto, SP, Brazil.; b Institute for Collective Health, Universidade Federal da Bahia. Salvador, BA, Brazil.

**Keywords:** Child, Child development, Zika virus, Criança, Desenvolvimento infantil, Zika vírus

## Abstract

**Objective:**

To identify scientific knowledge about the attention to health surveillance and development of Brazilian children under the age of three years involving the Congenital Zika virus (ZIKV) Syndrome.

**Data sources:**

This is an integrative literature review of primary studies with Brazilian children under three years of age from 2015 to 2019. The searches were carried out in the databases Latin American and Caribbean Literature in Health Sciences (LILACS), US National Library of Medicine (PubMed), Cumulative Index to Nursing and Allied Health Literature (CINAHL), SCOPUS and Web of Science. It was carried out by crossing the keywords in English (child, child development and Zika virus) and in Portuguese (criança, desenvolvimento infantil e Zika vírus), with the combination of the Boolean operator “AND”.

**Data synthesis:**

The knowledge produced is related to the specific health and development problems of children affected by the Congenital ZIKV Syndrome, with clinical characteristics, care demands, multiprofessional performance, health monitoring and surveillance needs.

**Conclusions:**

This integrative review synthesized scientific knowledge by adding aspects that reinforce the relevance of appropriate approaches to assess and care for children, linked to the engagement of caregivers, the need to document, evaluate and track the situations of children in early childhood and long-term, management coordination of care and its challenges in the context of primary health care.

## RESUMO

**Objetivo:**

Identificar os conhecimentos científicos sobre a atenção à vigilância à saúde e o desenvolvimento de crianças brasileiras menores de três anos envolvendo a síndrome congênita do Zika vírus.

**Fontes de dados:**

Trata-se de uma revisão integrativa da literatura, do período de 2015 a 2019, de estudos primários com crianças brasileiras menores de três anos de idade. As buscas foram realizadas nas bases de dados Literatura Latino-Americana e do Caribe em Ciências da Saúde, National Library of Medicine, Cumulative Index to Nursing and Allied Health Literature, Scopus e Web of Science. Foi realizado cruzamento das palavras-chave em inglês (*child*, *child development* e *Zika virus*) e em português (criança, desenvolvimento infantil e Zika vírus), com a combinação do operador booleano *and*.

**Síntese dos dados:**

Os conhecimentos produzidos estão relacionados aos problemas específicos de saúde e desenvolvimento das crianças afetadas pela síndrome congênita do Zika vírus, com características clínicas, demandas de cuidados, atuação multiprofissional, necessidades de monitoramento e vigilância à saúde.

**Conclusões:**

Esta revisão integrativa sintetizou conhecimentos científicos adicionando aspectos que reforçam a relevância de abordagens adequadas para avaliar as crianças e cuidar delas, articulado ao engajamento dos cuidadores, à necessidade de documentar, avaliar e rastrear as situações das crianças na primeira infância e em longo alcance, à gestão coordenada do cuidado e aos seus desafios no contexto da atenção primária à saúde.

## INTRODUCTION

Health care for children with chronic conditions such as the syndrome caused by the Zika virus (ZIKV) is of great relevance, since there are different demands, information and guidelines for the care of the child and their family. The ZIKV outbreak, which hit Brazil in 2015 and left the world on alert, represents a serious public health problem because of the degree of impairment that the congenital ZIKV syndrome causes, with a rapid dispersion capacity^1^ and a continuum of consequences for the children and their families.^2^

Since then, scientific evidence points to the causal relationship between ZIKV and defects in babies, causing miscarriages, early mortality, congenital microcephaly and neurological syndromes such as Guillain-Barré, meningoencephalitis and encephalomyelitis,^1^ ^,^ ^3^ not to mention the presence of ZIKV in the amniotic fluid in pregnant women and in the brain tissue of newborns with microcephaly, and the viral genome in the placenta of an intrauterine fetus.^4^ Thus, the ZIKV began to be associated with the large number of cases of microcephaly and other severe brain malformations in children.

The first generation of families with children affected by the congenital ZIKV syndrome suffered from the helplessness of government agencies and with care gaps for the limitations that babies had and family members faced.^5^ Monitoring the child’s health is vital when identifying vulnerability situations and for the intervention with actions of stimulus and attention to the demands of care, since these are children with special and complex needs^6^ ^,^ ^7^ and whose families encounter substantial challenges.^8^

In view of the impact of the congenital ZIKV syndrome, it is worth emphasizing the relevance of the difficulties, needs and demands of care and surveillance to the health and development of these children and the support to their family. This study aimed to identify scientific knowledge about the attention to health surveillance and the development of Brazilian children under the age of three years affected by the congenital ZIKV syndrome.

## METHOD

This is an integrative literature review, designated as a strategy to review scientific publications and synthesize its results, providing information and aiming to broaden the understanding of a given theme.^9^ ^,^ ^10^

In the first stage, in order to assist in the elaboration of the research question, the PICO strategy was used,^11^ ^,^ ^12^ acronym that represents: patient (P) - children under three years of age with congenital ZIKV syndrome; intervention (I) - child health development and surveillance; outcome (CO) - scientific knowledge about the care of children with congenital ZIKV syndrome from the perspective of health surveillance, development and its challenges. Thus, the guiding question of the study was: “What is the scientific knowledge about the development and health surveillance of children under three years of age affected by the congenital ZIKV syndrome?”.

To identify publications, the databases of Latin American and Caribbean Literature in Health Sciences (LILACS), National Library of Medicine, Cumulative Index to Nursing and Allied Health Literature, Scopus and Web of Science were selected. To search for publications, we crossed the keywords in English “child”, “child development” and “Zika virus”, and in Portuguese “*criança*”, “*desenvolvimento infantil*” and “*Zika virus*”, with the combination by the Boolean operator “and” (restrictive combination).

The inclusion criteria were publications written in Portuguese and in English, from 2015 to 2019, including primary studies with Brazilian children under three years of age. Literature reviews, websites, books, book chapters, theses, dissertations, booklets and magazine materials were excluded.

**Figure 1.**
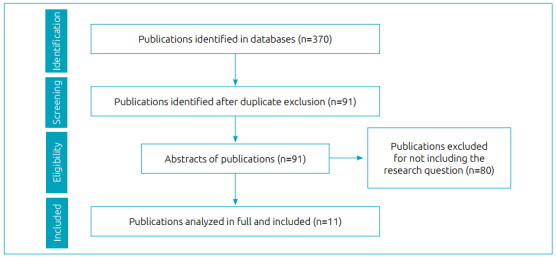
Flowchart of the literature search strategy, based on Moher et al.^13^.

The search for publications and selection were conducted in February 2020 by three authors and aided by the Rayyan^®^ software. Figure 1 shows a flowchart of the search strategy in the literature based on Moher et al.^13^

370 studies were identified in the listed databases. Then, 279 references were removed with the use of a tool to remove duplicates, resulting in 91 studies selected for reading of titles and abstracts. After the reading, 80 studies were excluded because they did not meet the inclusion criteria: 31 were not directed at children and the impact on their growth and development process; 15 brought other issues related to the virus; 11 were not cases of Brazilian children; 9 were only about chikungunya and/or dengue; 7 studies dealt with other viruses unrelated to the *Aedes aegypti* mosquito; 5 were literature reviews; 1 study had been published in 2020; 1 study was aimed at mothers and did not inform the children’s age.

With this strategy, 11 publications were selected to the stage of full reading. All of these met the research criteria and made up the present integrative review.

## RESULTS

The studies included^14^ ^,^ ^15^ ^,^ ^16^ ^,^ ^17^ ^,^ ^18^ ^,^ ^19^ ^,^ ^20^ ^,^ ^21^ ^,^ ^22^ ^,^ ^23^ ^,^ ^24^ are all scientific productions with Brazilian children published between 2016 and 2019. The general characteristics of the selected studies are shown in Chart 1.

**Chart 1.** Characteristics of studies included in the integrative review, 2020.

Authors/year	Title	Study design	Children’s age group	Local
Silva et al., 2016^14^	Early growth and neurologic outcomes of infants with probable congenital zika virus syndrome	Descriptive study	48 children from 1 to 8 months old	São Luís-MA
Botelho et al., 2016^15^	Presumed congenital Zika virus infection: findings of neuropsychomotor development - case report	Case report	4 children from 0 to 4 months old	Recife-PE
Satterfield-Nash et al., 2017^16^	Health and development at age 19-24 months of 19 children who were born with microcephaly and laboratory evidence of congenital zika virus infection during the 2015 zika virus outbreak - Brazil, 2017	Descriptive study	19 children from 19 to 24 months of age	Campina Grande-PB and João Pessoa-PB
Freire et al., 2018^17^	Congenital Zika virus syndrome in infants: repercussions on the promotion of mental health in their families	Qualitative study with participant observation	20 children from 2 to 8 months old	Rio de Janeiro-RJ
Wheeler et al., 2018 ^18^.	Skills attained by infants with congenital zika syndrome: pilot data from Brazil	Exploratory descriptive study	47 children from 13 to 22 months old	Recife-PE
Carvalho et al., 2018^19^	Congenital zika virus infection with normal neurodevelopmental outcome, Brazil	Case report	1 child from 0 to 20 months old	Salvador-BA
Alves et al., 2018^20^	Neurodevelopment of 24 children born in Brazil with congenital zika syndrome in 2015: a case series study	Descriptive study	24 children from 16 to 24 months of age	Recife-PE
Silva et al., 2019^21^	The frevo step enhancing the rehabilitation of children with congenital Zika virus syndrome	Descriptive study	30 children aged 9 to 18 months	Recife-PE
Lima et al., 2019^22^	Analysis of the functional performance of infants with congenital Zika syndrome: a longitudinal study	Longitudinal study	16 children from 6 to 24 months of age	Recife-PE
Einspieler et al., 2019^23^	Association of infants exposed to prenatal zika virus infection with their clinical, neurologic, and developmental status evaluated via the General Movement Assessment Tool	Cohort study	444 children from gestation to 12 months of age	Rio de Janeiro-RJ e Belo Horizonte-MG
Faiçal et al., 2019^24^	Neurodevelopmental delay in normocephalic children with in utero exposure to zika virus	Descriptive study	29 children 18 months to 22 months old	Salvador-BA

The publications addressed the situation in which the children’s health is affected by the congenital ZIKV syndrome, and the results of our review were organized in the following thematic units:

- Clinical specificities of the congenital ZIKV syndrome: monitoring of children’s characteristics;
- Demands of lifelong care and multidisciplinary approach;
- Gaps and benefits of health surveillance for children affected by the congenital ZIKV syndrome.

### Clinical specificities of congenital Zika virus syndrome: monitoring of children’s characteristics

The clinical specificities in children affected by the congenital ZIKV syndrome involve differential characteristics that were related to severe microcephaly and rupture of the fetal brain, with craniofacial disproportion, biparietal depression, occipital prominence, excessive scalp skin, and imaging tests showing cerebral calcifications and cortical malformations.^14^ The aforementioned abnormalities in the central nervous system bring specificities to the children’s development process, especially the ones with differential characteristics, who present seizure, dysphagia, irritability, hypertonia, hyperreflexia and increased primitive reflexes.^14^ A study^15^ also reported children who had in common hyperreflexia, hypertonia, atypical development and deficit in manual function, but vision and swallowing did not follow the same patterns, suggesting an association with brain changes.

In addition, non-febrile seizures have been described, indicating seizure disorder, hospitalization for bronchitis and pneumonia, difficulty sleeping, eating or swallowing, severe motor impairment compatible with cerebral palsy, impaired response to auditory stimuli, abnormalities in the retina and impaired response to visual stimuli.^16^

Children who were exposed to ZIKV while still in the uterus were followed up and presented delays in neurological development,^24^ which leads us to consider that the assessments should be early and detailed in order to detect neurodevelopmental problems.

Children who required hospitalization until the age of two were part of a study^20^ that found the reasons for hospitalizations, including uncontrolled epilepsy, need for ventriculoperitoneal shunting, infection, diarrhea, urinary tract infection and pneumonia, as well as recurrent episodes of seizure and use of antiepileptic drugs.

Neurological assessment of children between three and four months of age showed atypical performance, with altered muscle tone, motor, sucking, swallowing and breathing skills.^15^ There were also cases of babies who had clubfoot, arthrogryposis and cleft palate.^14^ Children between 18 and 24 months of age showed impaired neuropsychomotor development, with repercussions on walking and language acquisition, not being able to be left be alone, walk or speak, which are expected milestones of development for this age group.^20^

In a study conducted with76 children without congenital microcephaly and 35 with microcephaly, the children affected by the defect had bilateral spastic cerebral palsy and no normal movement.^23^ Among 47 children between 13 and 22 months of age evaluated at 16 months, none of them had the expected developmental skills for their age, and sleep was considered a problem for about 18% of the sample.^18^ In another publication including 48 infants, 85.4% had irritability, which became the most common symptom described, followed by pyramidal/extrapyramidal syndrome (56.3%), epileptic seizures (50.0%) and dysphagia (14.6%).

Children with neuropsychomotor disorders have difficulties in routine activities, in self-care and even to play, as shown in a study that reported that the findings identified at birth remain present in the age group of 19 to 24 months.^16^ In clinical examinations of children with a mean age of 19.9 months, the age equivalent to 2.1 months were found for language development, 2.7 months for gross motor activity, 3.1 months for fine motor activity, and 3.4 months for adaptative/social activity.^20^ Another study with children between 6 and 24 months found delay in functional development, with a slow longitudinal evolution in the age range evaluated.^22^

Children exposed to the ZIKV and who did not have microcephaly had their development compromised.^23^ More specifically, children appeared to have relative strengths in communication and gross motor skills, while weaknesses were found in their fine motor skills.^18^

### Child and family care demands and multiprofessional approach

Care demands for children affected by the congenital ZIKV syndrome are related to clinical manifestations, with challenges in feeding, sleep, neuromotor, socioemotional, visual, auditory and language development due to difficulties and abnormalities that can occur concurrently.^16^

Many of the initial discoveries identified at birth remain present between 19 and 24 months of age, and these children are lagging behind in reaching age-appropriate developmental milestones, and this indicates the need for long-term follow-up and support.^16^ The provision of initial information about possible deficits in children’s development is highlighted as fundamental, aiming to assist the rehabilitation team in early intervention and, consequently, to minimize future functional limitations.^15^

The need for a connection between the multidisciplinary team and the family is reinforced for the sharing of practices and knowledge based on social and socio-emotional support, and, when it comes to the psychic impact, it has been shown that there is a need for welcoming and mental-health promotion guidelines aimed at the family demands.^17^

In a study on the evaluation of a multiprofessional team with families, the use of social networks was reported as a positive aspect, a means for communication and discussion of different experiences and sharing of strategies to overcome diagnosed impossibilities.^17^ In another study, we used costumes and photographs to facilitate parents’ interaction and understanding of therapeutic guidelines, confirming that adopting cultural-context elements is favorable and beneficial and should be used by the occupational therapist.^21^

The need for timely intervention services and resource planning to support families in health services and communities is reinforced.^16^

### Gaps and benefits of monitoring and health surveillance of children affected by congenital Zika virus syndrome

The context of a child’s health involving the congenital ZIKV syndrome and post-ZIKV infection consequences point to the need for surveillance of signs and symptoms presented. The need for a detailed assessment stands out, even for children in typical development with possible congenital ZIKV infection, who receive care at a later stage,^19^ suggesting gaps that need to be identified and monitored.

When it comes to the monitoring of children’s health, a publication on early stimulation in cases of congenital ZIKV syndrome reported the recognition of this strategy by mothers as beneficial for the child’s development, although difficulties in transportation to access health services and assistance were also found.^21^

The high specificity of the General Movement Assessment tool was considered useful, with a view to assisting in issues that involve limiting the unnecessary referral of children to highly overburdened rehabilitation services, as well as facilitating immediate referral to these services when needed, as it can optimize development, prevent secondary complications and improve family well-being.^23^

Child health surveillance is seen by the perpective of need for early prognostic markers,^19^ which points to the relevance of investigating suspected cases of ZIKV infection, regardless of whether child neurodevelopment is showing normal characteristics or not.

During pregnancy and for children who did not have microcephaly, health monitoring and surveillance are also relevant, since a routine assessments enables early entry into more targeted intervention programs if needed.^23^

The identification of developmental milestones not compatible with age is highlighted as important in anticipating the needs for health services and social programs for children affected and their families, including early intervention services, resource planning, monitoring and surveillance actions aimed at their growth and development process.^16^

Therapeutic follow-up was considered indicative of the need for public policies that strengthen long-term follow-up strategies for children.^22^ Support for parents in this trajectory was emphasized, meeting the different demands arising from the congenital ZIKV syndrome, including family members mental health and the promotion of actions that can favor the child’s development.^17^

The systematic assessment of children in situations of health impairment involving the congenital ZIKV syndrome, using standardized tools,^24^ is crucial for the early detection of developmental problems. This intervention is recommended to achieve better results and prevent other deficiencies, which suggests gaps to be monitored and investigated.

## DISCUSSION

The present study identified the knowledge produced in the scientific field related to specific health and development problems of children affected by the congenital ZIKV syndrome, with clinical characteristics, care demands, multiprofessional performance, health monitoring and surveillance needs.

Children who developed congenital ZIKV syndrome had chronic physical, developmental, behavioral or emotional deficits that demand care and assistance from different services and supports^25^ throughout their lives, including their family, the multidisciplinary team and Other health fields, considering they have special needs. The findings that suggest that babies exposed to ZIKV and asymptomatic at birth may develop problems later also suggest the need for more assessments and neurological, neurodevelopmental, neurobehavioral, auditory and visual management, since lessons have already been learned from other congenital infections, with valuable clues about the complex conditions that affect these children’s health.^26^

The demand for care and attention focused on the development of the child affected by the ZIKV is approached as continuous and must be redoubled in a process that implies confrontations and challenges for professionals and families,^8^ as there is a range of needs and treatment options should be made available. Rearrangements of care demands with families are important for the actions of professionals from different services.^27^

The creation of therapeutic groups aimed at parents, caregivers and babies was also pointed as beneficial in ensuring access to a social support network,^27^ in order to assess the progress and individuality of each child’s development and guidelines related to monitoring and daily care. The relevance of care coordination,^8^ the performance of multiprofessional health teams, school and legal services is highlighted in this context.^28^

The lack of specific treatment for microcephaly is pointed out,^29^ but the disease requires special care. In the health system, the need for adaptation of the effective care of children and their families is shown, with support and training for teams in different specialties.^30^

The process of development and growth associated with possible health problems that can be caused by the congenital ZIKV syndrome should be further analyzed in order to bring scientific evidence that increases and sustains care towards the integral care of children and their families. Thus, the care needed in the family also needs to be explored, considering that the family is fundamental in facing situations and building positive problem solving.^7^

In relation to action plans that encompass children and families with extended professional teams, the organizing axes are emphasized around the diagnostic, etiological and tracking of children’s developmental problems,^31^ which requires research on emotional impact, quality of life, family coping strategies and specific training for multiprofessional teams. Still, the early-intervention professional must understand the ZIKV infection, including geographic risk, etiology and epidemiology, based on emerging scientific information and the impairments not yet known.^28^

Over time, particularly after the 2015 and 2016 cases in Brazil, the follow up of children will bring more support and scientific evidence about the consequences of the disease, the implications of the infection in certain gestational periods and the longitudinal analyzes of clinical outcomes.^28^ ^,^ ^32^

Different professionals are engaged in discussions on long-term surveillance and therapeutic measures, with the purpose of being closer to the care of children with the congenital ZIKV syndrome and their families.^18^ Professionals initially in need of more knowledge about the disease were pediatricians, as they are involved in the investigation of microcephaly.^33^ However, all health professionals must be aware of the social stigma linked to this congenital infection and its additional implications, with special attention to environments, characteristics and situations of vulnerability of the child and family, with possible negative results, including abuse and abandonment;^34^ that way, they can provide assistance to the child, their family and the community, and also perform health surveillance in primary health care.

Family members are considered a fundamental part of the daily care of the child and are seen as protagonists for effective stimulation.^35^ Thus, advice to families and support to parents are pointed out by government agencies as extremely important,^29^ as well as aspects about the gradual construction of the emotional bond with the child to favor their development and quality of life within the limitations they present,^36^ taking into account that each child has their own pace and needs longitudinal monitoring.^37^ Attention with timely interventions should be part of the support to the mental health of parental caregivers in early intervention programs for the development of children and families.^38^

The situations involving the congenital ZIKV syndrome also have implications in the construction of parenting, which requires different family dimensions and sectors of society. In this context, the role of the nursing professional is relevant, as they are a privileged point of contact with the child and their families in different levels of care.^39^

Thus, it is up to health professionals, each in their field of knowledge, to provide care for families and carry out appropriate interventions, so that they can understand the continuous care needs inherent to the conditions of children affected by the congenital ZIKV syndrome.

Another aspect to note is that diseases with complex patterns of transmission, encompassing environmental, social, economic, or even unknown determinants, and those transmitted by insect vectors are considered difficult to control,^30^ like chronic diseases with infectious periods that demand long periods of treatment. Early childhood is the period that encompasses pregnancy until three years of age, a crucial stage for the improvement of brain structures and human development.^40^ ^,^ ^41^ Interventions in prenatal care and in the early years are essential for life, providing health and well-being benefits and minimizing one’s vulnerability to potential environmental harmful effects.^42^

The limitations of this study refer to procedures for the use of the Boolean operator “and” instead of “or” in searches between descriptors, which retrieved more than a thousand studies per database and made a detailed and judicious analysis impossible. Primary studies addressing the health of children with the congenital ZIKV syndrome were included, but some may have been excluded by the referred research procedure with relevant scientific evidence interrelated to the theme, which also contributed with other elements for the synthesis of the present review.

This integrative review synthesized the scientific results with information and amplitude of the theme, bringing aspects that reinforced the relevance of appropriate approaches to assess and care for children, linked to the engagement of caregivers and the need to document, evaluate and track children’s situations in early childhood and in a long-range, as well as the importance of coordinated care management and its challenges in primary health care.

The actions of stimulating the child with guidance to relatives and attention to the demands of care with the participation of multidisciplinary teams were the most emphasized recommendations in the studies of this integrative review. The scientific literature shows that the congenital ZIKV syndrome is still an emerging topic. The results of this investigation suggest that caring for children who were born with microcephaly and/or developed the congenital ZIKV syndrome requires deepening and continuing surveillance of health and development goals. Thus, other investigations may bring more contributions and scientific evidence for the assessment and monitoring of different situations and vulnerabilities of children and their families.
